# Mercury-Pollution Induction of Intracellular Lipid Accumulation and Lysosomal Compartment Amplification in the Benthic Foraminifer *Ammonia parkinsoniana*

**DOI:** 10.1371/journal.pone.0162401

**Published:** 2016-09-07

**Authors:** Fabrizio Frontalini, Davide Curzi, Erica Cesarini, Barbara Canonico, Francesco M. Giordano, Rita De Matteis, Joan M. Bernhard, Nadia Pieretti, Baohua Gu, Jeremy R. Eskelsen, Aaron M. Jubb, Linduo Zhao, Eric M. Pierce, Pietro Gobbi, Stefano Papa, Rodolfo Coccioni

**Affiliations:** 1 Department of Pure and Applied Sciences, Urbino University, Urbino, Italy; 2 Department of Biomolecular Sciences, Urbino University, Urbino, Italy; 3 Geology and Geophysics Department, Woods Hole Oceanographic Institution, Woods Hole, MA, United States of America; 4 Environmental Sciences Division, Oak Ridge National Laboratory, Oak Ridge, TN, United States of America; Chinese Academy of Sciences, CHINA

## Abstract

Heavy metals such as mercury (Hg) pose a significant health hazard through bioaccumulation and biomagnification. By penetrating cell membranes, heavy metal ions may lead to pathological conditions. Here we examined the responses of *Ammonia parkinsoniana*, a benthic foraminiferan, to different concentrations of Hg in the artificial sea water. Confocal images of untreated and treated specimens using fluorescent probes (Nile Red and Acridine Orange) provided an opportunity for visualizing the intracellular lipid accumulation and acidic compartment regulation. With increased Hg over time, we observed an increased number of lipid droplets, which may have acted as a detoxifying organelle where Hg is sequestered and biologically inactivated. Further, Hg seems to promote the proliferation of lysosomes both in terms of number and dimension that, at the highest level of Hg, resulted in cell death. We report, for the first time, the presence of Hg within the foraminiferal cell: at the basal part of pores, in the organic linings of the foramen/septa, and as cytoplasmic accumulations.

## Introduction

Foraminifera are single–celled organisms (protists) with a shell (i.e., test) that may be organic, agglutinated, composed of secreted calcium carbonate or, in rare cases, silica [1]. They are considered one of the most diverse group of shelled microorganisms in modern oceans [2] and play a significant role in global biogeochemical cycles of inorganic and organic compounds [3]. Traditionally, the study of foraminifera has been the domain of paleontologists that have used them for petroleum exploration surveys, and palaeoclimatological and palaeoecological reconstructions. Benthic foraminifera have also been used as bioindicators of pollution in a variety of marine and transitional marine environments (i.e., [4–5]). They respond to adverse ecological conditions including pollution by changing assemblage compositions and parameters (diversity and density) as well as cellular ultrastructure (reviewed in [6–7]). Despite major advances on foraminiferal ecology and biology that have been achieved over 50–60 years, the ultrastructure-induced changes due to pollution [8–11] are not fully understood. An increase of lipid droplets (LD) characterized by a more electron-dense core, proliferation of residual bodies, a thickening of the organic lining, mitochondrial degeneration and autophagosome proliferation are among the main cytological alterations of benthic foraminifera in response to pollutant exposure [9–11].

The proliferation of fibrillar and large lipidic vesicles, their possible exocytosis and an increase in the number of residual bodies have been documented in two *Ammonia* species after exposure to different copper (Cu) concentrations [10]. Interestingly, those authors reported the presence of larger lipid vesicles in all chambers of specimens exposed to higher Cu; these results were interpreted as a perturbation in the foraminiferal metabolic regulation due to pollution. The thickening of the organic lining might be ascribed to fibrillar material provided through fibrillar vesicles that discharge their content by exocytosis (i.e., [7,12]). It was suggested that the thickening of the organic lining might have a defense mechanism against pollutants [6–7]. A higher number of LD was noted in *Ammonia parkinsoniana* exposed to high concentrations of lead (Pb); these LD were also characterized by an electron-dense core that was not, however, visible in the LD from control specimens [11]. Experiments on *Rosalina leei* revealed that increasing concentrations of Hg promote the inhibition of reticulopodial activity, and more importantly, the development of test abnormalities as well as a reduction of growth [13–14]. Despite these observations, no ultrastructural studies have been performed on benthic foraminifera exposed to Hg. Mercury and most of its compounds are extremely toxic and are considered among the most harmful toxins for living organisms [15–16]. Mercury is known to bioaccumulate and biomagnify as methylmercury (CH_3_Hg^+^) in aquatic organisms [17].

The aim of this contribution was, therefore, to assess the variation in the distribution and amount of lipids in *A*. *parkinsoniana* when exposed to different concentrations of Hg and to localize the presence of this pollutant within the cytoplasm. This was achieved using transmission electron (TEM), environmental scanning electron (ESEM), and scanning electron (SEM) microscopies coupled with energy dispersive X-ray spectrometry (EDS), and confocal laser scanning microscopy (CLSM) with fluorescent dyes.

## Materials and Methods

### Sediment sampling and laboratory mercury treatment

Sediment samples were collected at 14-m water depth off the Monte Conero area (Italy, Adriatic Sea). The collection site (43°33'54'' N, 13°39'52'' E) is in a coastal area located close to the terrestrial Regional Natural Park of Conero characterized by little human activity, with largely diversified benthic foraminiferal assemblages and oligo-mesotrophic conditions [18]. No permits or approvals were required to collect sediments at this site. No threatened or endangered species were involved. At the collection site, temperature, pH, salinity, Eh and dissolved oxygen of seawater were measured in vertical profile using a multiparameter CTD (Conductivity, Temperature and Depth) probe (Hydrolab, MiniSonde 4a). Sediment was sampled by multiple deployments, at the same station, of a Van Veen grab sampler that collects sediment over a surface area of about 400 cm^2^ and only the surface 2 cm were retained. On board, the sediment was thoroughly homogenized and subsequently sieved over a 500-μm screen with ambient seawater. The >500-μm fraction was discarded to remove potentially disturbing effects of bioturbators (i.e., macrofauna and large meiofauna). The <500-μm fraction, which bore foraminifera, was placed in an insulated box, covered with ambient seawater, and kept near ambient temperature until arrival at our shore-based laboratory. The study did not involve any animal (animals, embryos or tissues) or human (human participants and/or tissue) participation.

Artificial Sea Water (ASW), which was prepared at in-situ temperature following the methods of [19], was stored in the dark and oxygenated. Two Hg-ASW mixture concentrations plus one control (i.e., ASW without added Hg) were prepared. The inorganic salt of Hg, as mercury chloride (HgCl_2_; >99.5% pure; CAS Number 7487-94-7; Sigma-Aldrich), was used for the experiments. The final pollutant concentrations for experimental media were obtained by adding appropriate volumes of stock solutions to ASW. High concentrations of Hg, namely 1 ppm and 100 ppm, were considered for the experiment to ensure that contaminant permeated the sediment and, in turn, incubated the benthic foraminiferal assemblages. Each tank (aquarium) (60 cm × 40 cm × 20 cm) was filled with 20L of Hg-ASW mixture. Mesocosms (15 cm × 8 cm × 3 cm) containing 1 cm-thick sediment were placed inside each tank. Multichannel pumps were used to circulate and to oxygenate water through silicone rubber tubing anchored between the tanks’ bottom and plastic grids. Tanks were placed in a controlled environment with air temperatures of 14–16°C that were uniformly maintained throughout the experiment. The dissolved oxygen (DO), salinity (S), conductivity, temperature (T), Oxidation Reduction Potential (ORP) and pH of the seawater were routinely monitored by a set of HQ40d (Hach Lange) portable multi-parameter probes [11]. Physico-chemical parameters (DO, S, T, pH and ORP) of seawater were kept mostly constant throughout the experiments. The mean value of salinity registered during the experiment was 36.98‰ with only a slight steady increase (<1‰). The DO in the tanks remained stable at ~9.5 mg/l through the experiment.

### Mesocosm experiment

One mesocosm was extracted at pre-established time intervals: two months (T1) and three months (T2) from each tank, namely control (c) and two selected concentrations of Hg-pollutants (1 ppm and 100 ppm). The 120-cm^3^ sediments from each mesocosm were wet-sieved with ASW through a 125-μm screen. The >125-μm fraction was the source material from which living specimens of *A*. *parkinsoniana* were picked. The presence of reticulopodial activity or, if absent (T2-100 ppm), the presence of cytoplasm in any but the last chamber was the criterion for specimen selection. The presence of cytoplasm in specimens of the T2-100 ppm group was not a sufficient condition for determining specimen viability so further analyses (ultrastructural investigations and confocal-cell imaging) were performed to evaluate cell fitness.

### Microscopic analyses

#### Transmission Electron Microscopy

Selected specimens were fixed with 2.5% glutaraldehyde (TAAB, England, UK) in ASW for 3 h at 4°C. Foraminiferal tests were then decalcified with 0.1 M EDTA for 36 h. After 5 washings with ASW, foraminiferal specimens were post-fixed with 1% osmium tetroxide (OsO_4_; EMS, Hatfield, PA) in ASW for 2 h at room temperature (RT). Following 5 washings, specimens were dehydrated in a graded series of ethanol baths, from 50% to 100% and immersed in propylene oxide (EMS, Hatfield, PA) twice, each for 10 minutes. Subsequently, they were embedded in epoxy resin by using increasing concentration of resin (Durcupan Araldite, SIGMA, UK). Foraminifera were ultimately sectioned using an ultramicrotome (LKB, 2088 UltrotomeV). Thick sections of 1 μm were stained with 1% toluidine blue in distilled water at 60°C to provide light-microscope-level overviews of whole sections. Thin sections (100 nm), collected on 300-mesh nickel grids, were stained with uranyl acetate and lead citrate and finally observed with a Philips CM10 transmission electron microscope at 80 KV [20]. Lipid was quantified by calculating the area of 200 lipid vesicles, on average, distributed in the last whorl excluding the final two chambers (last two formed; i.e., youngest two) from 3–6 selected specimens (200–350 μm in diameter) for the two Hg concentrations, plus controls, at both time points. The Mann-Whitney U test, a nonparametric test, was used to check for significant differences between mean lipidic dimension among experimental conditions (concentration and time).

#### Confocal and light microscopy

Selected specimens of *A*. *parkinsoniana* were incubated with Nile Red (NR) or Acridine Orange (AO) at T1 and T2 and then analyzed with CLSM.

Nile Red is a phenoxazine dye used on living and fixed cells to localize and quantify neutral and polar lipids [21–22]. The absorption and fluorescence properties of NR are known to be sensitive to environmental factors such as polarity. Polar lipids (i.e., phospholipids), which are mostly present in membranes, fluoresce red (emission > 590 nm) whereas neutral lipids (esterified cholesterol and triglycerides), which are present in LD, fluoresce yellow (570–590 nm) [23–24]. NR was used on specimens of *A*. *parkinsoniana* to detect membranous vesicles and on whole specimens to compare the lipidic distributions of untreated and Hg-treated conditions by confocal microscopy. For NR microscopy, *A*. *parkinsoniana* specimens were fixed in 2% paraformaldehyde for 2 hours, then washed in ASW and decalcified with EDTA (0.1 M) for 48 hours to remove the foraminiferal test. Following decalcification, specimens were rinsed in ASW, transferred to MatTek glass bottom chambers (MatTek Corporation, Ashland, MA) and NR was added at the final concentration of 3 μg/ml for 40 min at RT. Using CLSM, specimens were subject to blue excitation (488 nm) and analyzed separately for yellow and red emissions. Quantitative analyses of NR Mean Fluorescence Intensity (MFI) were performed using ImageJ software (NIH, Bethesda, MD), imaging the specimens at the same magnification, and determining the MFI of all the selected pixels. Subsequently, yellow MFI values were converted to arbitrary units (A.U.) setting the first image of the first control specimen at 100. The fluorescence emission of LD was calculated in the yellow channel of 3–5 selected specimens (200–350 μm in diameter) for each concentration.

The pH-sensitive dye acridine orange (AO) was used to detect and quantify acidic vesicular organelles in *A*. *parkinsoniana* specimens by confocal microscopy [25–26]. AO is a cell-permeable fluorescent dye that labels DNA and cytoplasm bright green whereas RNA and acidic vacuoles appear red. It can also enter acidic compartments and organelles, such as lysosomes and autolysosomes, where it becomes protonated and sequestered [25]. When AO is bound to acid compartments, such as lysosomes and acidic vacuoles, it emits red fluorescence (>650 nm) with intensity proportional to the acidity degree. For confocal live imaging, *A*. *parkinsoniana* specimens were transferred to MatTek glass bottom chambers (MatTek corporation, Ashland, MA) and then stained with AO (150 ng/ml) and incubated for 40 minutes at RT. Green and red fluorescence emissions illuminated with blue (488 nm) excitation light were analyzed by confocal microscopy. Because AO must be used on living specimens and *A*. *parkinsoniana* tests may interfere with dye uptake, only peripheral signals were observed (cytoplasm in green and acidic vesicles in red). Quantitative analyses of AO red MFI were performed on 3–5 specimens (200–350 μm in diameter) for control and 100 ppm treatments. Image analyses were carried out by determining the red MFI of all the selected pixels of the imaged specimens. Subsequently, the red MFI was converted to arbitrary units (A.U.) setting the first image of the first control specimen at 100. Epifluorescence and bright field (BF) microscopies were performed using a CLSM (Leica TCS SP5 II confocal microscope, Leica Microsystems) with 488-, 543- and 633-nm illumination and oil-immersion objectives.

#### ESEM-SEM and EDS

Embedded specimens used for TEM were observed, as a whole, with an environmental scanning electron microscope (FEI ESEM, Quanta 200) to qualitatively characterize the presence of Hg. The ESEM, coupled with energy dispersive X-ray spectrometry (EDS), was used to assess the elemental composition of particles in the cytoplasm. The EDS is a technique employed to collect and determine the energy and the number of X-rays that are given off by atoms in a material [27–28]. Observations were conducted in low vacuum (0.2–1.2 Torr) at 10-mm working distance using secondary and backscattered electron modes with energy varying from 12 to 25 kV. A live counting time of 100 seconds, with spots’ mode from 3 to 5, was used for elemental mapping.

Additionally, thin sections (~100 nm) were mounted onto freshly peeled mica adhered to an SEM stub with double stick tape. The sections were then coated with about 10 nm of carbon using a Cressington 208 carbon coater. The sample was then imaged and elemental maps were obtained using a Hitachi S4800 FEG-SEM at 20 keV equipped with an EDS at the Oak Ridge National Laboratory. The upper secondary detector was used for image acquisition at a working distance of 7–8 mm. EDS maps were obtained using 64 sweeps with a 200 ms dwell time per pixel at a resolution of 256×200 pixels. Maps were plotted using the net intensities of the selected regions of interest with the nearest 3 × 3 pixels averaged to improve signal to noise in the element map images.

## Results

### Lipid characterization

**Fig 1:**
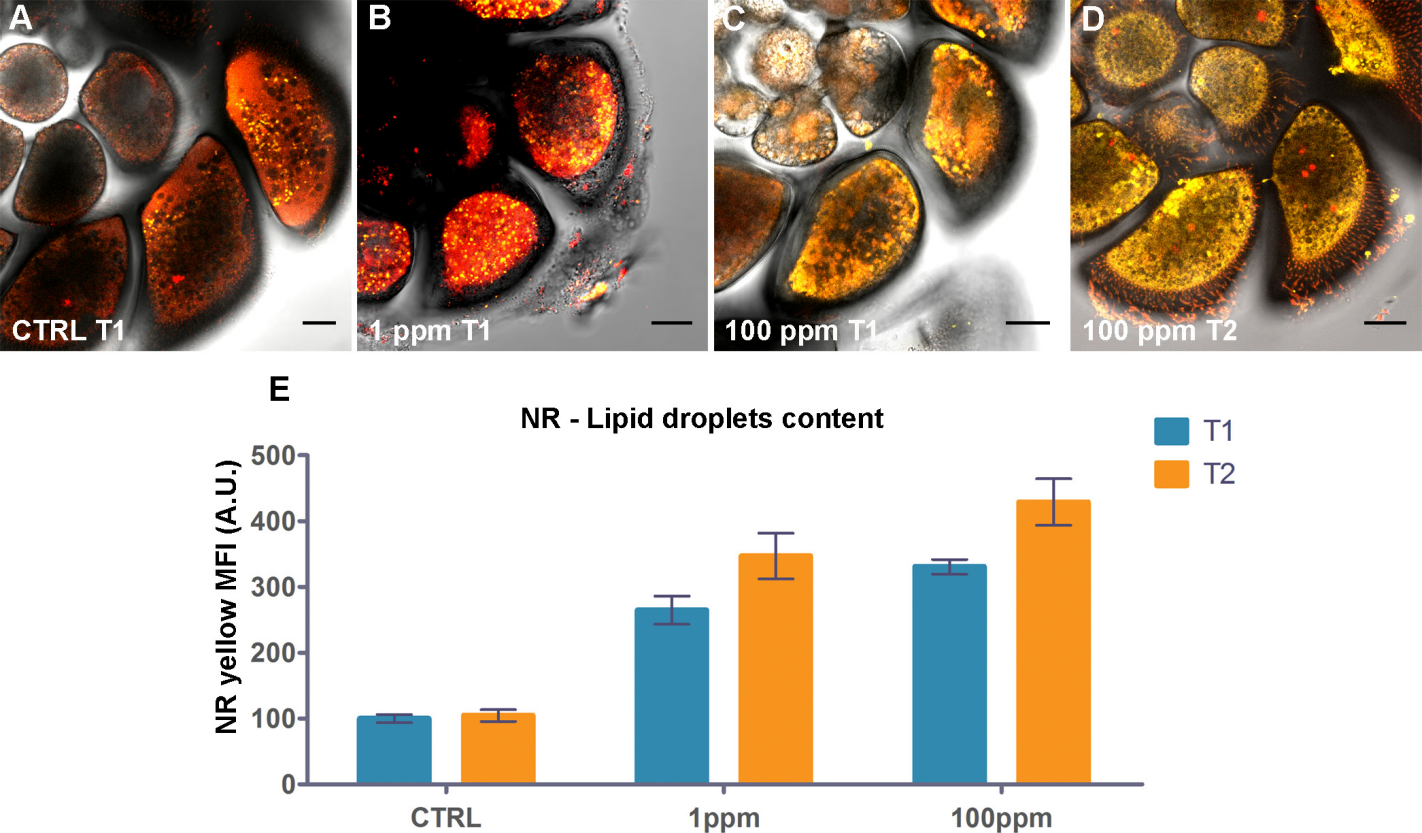
Effect of Hg exposure on lipid distribution of *A*. *parkinsoniana* labeled with NR. Epifluoresecence micrographs of single optical sections showing overlay of NR yellow and red fluorescence for (A) T1- control, (B) T1-1 ppm, (C) T1-100 ppm and (D) T2-100 ppm, Bars: 20 μm. (E) Histogram of yellow Mean Fluorescence Intensity (MFI) expressed in arbitrary units (A.U.) for the three treatments over time (control, 1 ppm, and 100 ppm at both T1 and T2). Error bars indicate ± standard error of the mean.

Images obtained visa CLSM showed enhanced accumulation of cellular lipids in Hg-treated *A*. *parkinsoniana* at T1 and T2, compared to control specimens (Fig 1A–1D). In untreated (control; no Hg exposure) *A*. *parkinsoniana* specimens, a compact and uniform lipid distribution was found (Fig 1A) whereas, at increasing Hg concentration at both T1 and T2, enhanced yellow fluorescence, in particular for the 100-ppm Hg treatment, was observed (Fig 1B–1D). NR quantification by ImageJ software confirmed this observation (Fig 1E), where NR yellow MFI markedly increased in Hg-treated specimens both in a time- and concentration-dependent manner. Negligible changes were observed between T1 and T2 among treatments (controls, 1 ppm, and 100 ppm). On the other hand, differences were evident between control and treatment, namely 1 ppm and 100 ppm at both T1 and T2 (Fig 1E).

Diameters from a total of 1295 LD from 27 specimens were measured from TEM images (S1 Appendix). On the basis of the Mann-Whitney U test, no significant differences in diameter were found among all the six conditions except for T2-100 ppm, which exhibited significantly larger LD (S2 Appendix).

### Lysosomal compartment characterization

**Fig 2:**
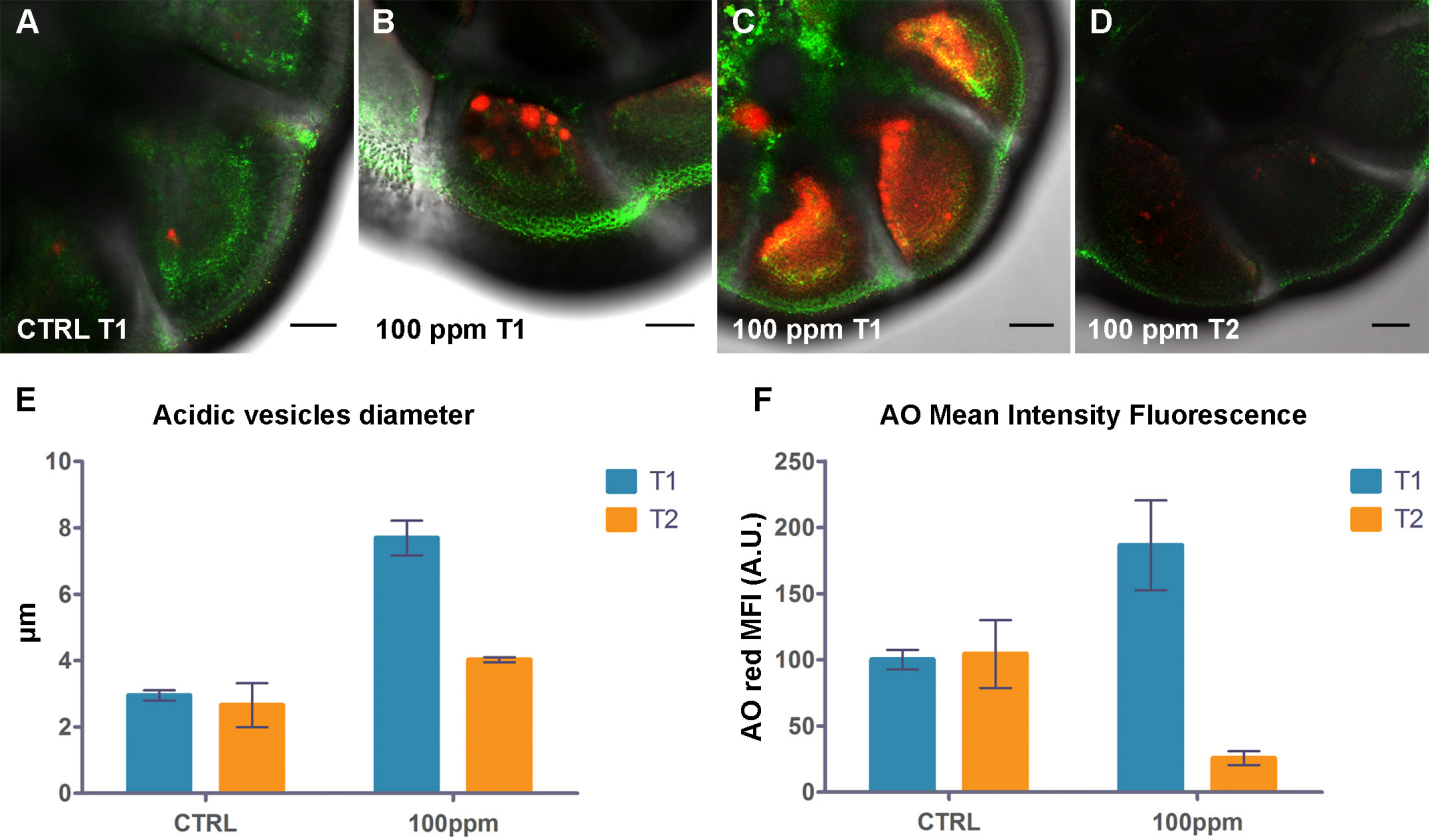
Effect of Hg exposure on acidic compartmentalization of *A*. *parkinsoniana* labeled with AO. Epifluorescence micrographs of single optical sections showing overlay of AO green and red fluorescence for (A) T1-control, (B,C) T1-100 ppm, and (D) T2-100 ppm, Bars: 20μm. (E) Histogram of maximum dimension (diameter) of acidic vesicles. (F) Histogram of red Mean Fluorescence Intensity (MFI) expressed in arbitrary units (A.U.) for control and 100 ppm at both T1 and T2. Error bars indicate ± standard error of the mean.

In order to evaluate the acidic compartments (lysosomes, autolysosomes and other acidic vesicular organelles) of *A*. *parkinsoniana* in response to Hg exposure, several specimens from each experimental condition were probed with AO (Fig 2A–2D). At T1, specimens exposed to 100-ppm Hg displayed more conspicuous acidic compartments, essentially due to vesicle volume (dimension and, as a consequence, total fluorescence intensity), when compared to those of control specimens (Fig 2E and 2F). Some 100 ppm-exposed specimens also exhibited diffuse red fluorescence in their chambers, suggesting cytoplasmic acidification (Fig 2C). These specimens were excluded from AO MFI quantitative analyses (Fig 2F). Mercury-exposed specimens at T2 appeared to show an acidic compartment decrease as marked by reduction of red vacuoles suggesting a loss of lysosomal activity, a possible signal of recent or imminent death (Fig 2D and 2F).

### Localization and distribution of Hg

**Fig 3:**
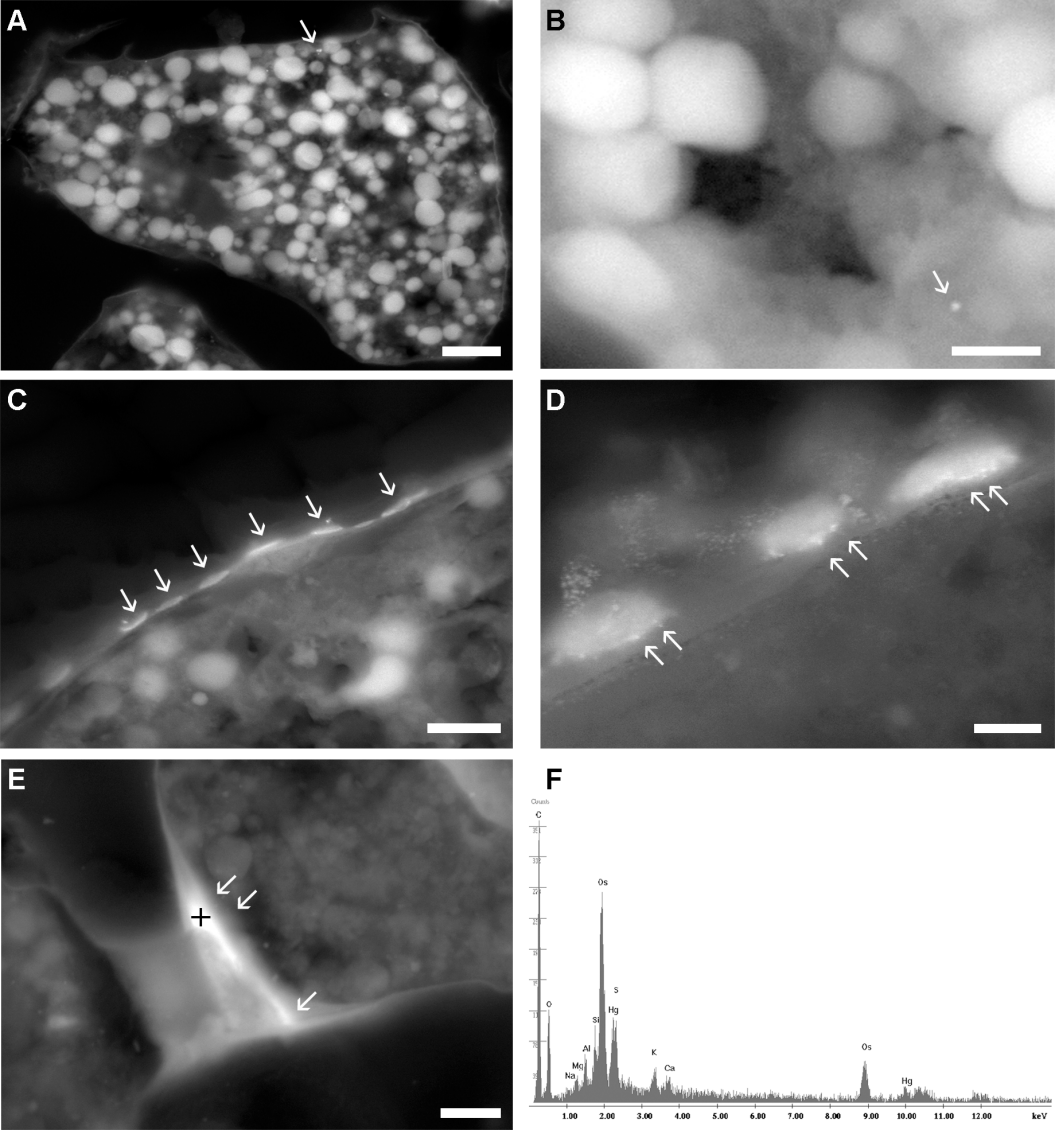
Micrographs showing presence of Hg in *A*. *parkinsoniana* specimens (T2-100 ppm). (A) young chamber containing vacuoles; (B) high magnification of a young chamber; (C,D) basal part of pores; (E) foramen/septum. (F) Example EDS spectrum taken with a spot size of 4 on cross (E). Arrows mark the occurrence of Hg. Scale bar: (A) 10 μm; (B) 1.5 μm; (C) 2.5 μm; (D) 0.8 μm; (E) 5 μm.

On the basis of ESEM-EDS observations, it was possible to observe the presence and distribution of Hg in different parts of the *A*. *parkinsoniana* (Fig 3). As expected, no Hg was found in control specimens. In Hg-incubated specimens, Hg was commonly found in all chambers (not shown) in the basal part of pores (Fig 3C and 3D) and in the foramen (Fig 3E), occurring as a diffuse signal corresponding to the organic linings. Mercury also occurred in the form of granules (1–2 μm) in the cytoplasm, mainly in the youngest chambers (Fig 3A and 3B). The elemental analysis revealed the presence of Hg in first and secondary peaks (Fig 3F). The bright large vacuoles (Fig 3A and 3B) in the cytoplasm appear to have a lipid nature as confirmed by EDS spectrum revealing the main presence of osmium. The presence of osmium was an artifact resulting from the fixation protocol for optical and electron microscopy, where it is commonly used to fix and to stain lipids.

The SEM-EDS element maps on thin sections confirm the presence of localized Hg in the basal part of the pores (S2 Fig). The Hg-rich region was observed as a lighter strip in the secondary emission (SE) SEM image (S2A and S2B Fig). The lighter contrast is a result of backscattered electrons from the heavier (higher atomic number) Hg atoms detected by the upper polepiece detector (S2C Fig). The element maps reveal the localized nature of the Hg on the outer lining just below the decalcified test region (S2D Fig). This is supported by the carbon depletion and calcium presence just to the left of the Hg strip. It is possible that the localized Hg could have been a HgS complex. However, since the EDS peak positions of the Hg M and the S K peaks overlap, it is difficult to exactly characterize the elemental composition. It is certain that this region contains significant amounts of Hg due to the brighter contrast compared to the background in the SE image (S2A–S2D Fig). Further studies are required to resolve these unknowns.

## Discussion

Heavy metals occur in ecosystems from both natural sources and human activities, with large variations in concentrations [29]. Some heavy metals are essential to life, but they can become toxic through bioaccumulation and biomagnification [30]. Membrane transport or passive diffusion of neutral chemical species are reported as pathways to incorporate heavy metals in cells [31]. Heavy metals might penetrate foraminiferal cell membranes together with food and/or may be incorporated from seawater by membrane transport [32]. Among heavy metals, Hg is considered to be highly toxic for living organisms [30]. Mercury is not biodegradable but bioaccumulates in the food web [33]. In particular, uncharged Hg species, including mercury sulfide (HgS), dimethylmercury ((CH_3_)_2_Hg), and inorganic mercury (HgCl_2_), are shown to cross cell membranes [34]. Methylmercury has been reported as the most toxic species of Hg because it can easily cross biological membranes; though inorganic Hg, which is capable of passing biological barriers with difficulty, is also toxic but at concentrations higher than methylmercury [35]. The concentration and form of Hg in aquatic organisms are controlled by passive uptake of lipophilic chloride complexes (both CH_3_HgCl and HgCl_2_) across the cell’s lipid bilayer in phytoplankton [36], whereas an active uptake of Hg(II) was suggested for iron-reducing bacteria (FeRB) *Geobacter sulfurreducens* and sulfate-reducing bacteria (SRB) *Desulfovibrio desulfuricans* (strain ND132) [37]. More importantly, in phytoplankton, Hg(II) is mainly bound to membranes whereas methylmercury is mostly accumulated inside the cell, in the cytoplasm [36, 38].

On the basis of different methodology, the concentrations of Hg used in our experiment were higher than those considered by [13, 14] that directly exposed the foraminifer *Rosalina leei* in culture and reported specimens living in concentrations up to 260 ng/l (260 ppt). Contrary to these culture experiments, the present study used higher Hg concentrations and also ensured that the Hg-ASW mixtures permeated the sediments to expose the foraminifera living therein. Although different culture experiments have been conducted to document the effect of heavy metal pollutants on foraminifera (i.e., [10, 13, 14]), to our knowledge, no study has ever revealed the direct presence of metals within the cytoplasm of foraminifera. The occurrence of Hg within the *A*. *parkinsoniana* cytoplasm suggests that the pollutant crossed the foraminiferal cell membrane in some manner. In particular, Hg was found in all foraminiferal chambers, from the oldest to the youngest and has been mainly found as small particles dispersed in the cytoplasm, and more importantly diffuse at the basal part of pores and along the organic lining.

The effects of pollutants on foraminiferal ultrastructure are not new to science. Indeed, numerical increases of LD characterized by a more electron-dense core, proliferation of residual bodies, fibrillar and large lipidic vesicles, a thickening of the organic lining, mitochondrial degeneration, and autophagosome proliferation are among the most common documented cytological modifications (i.e., [9–11]). Unfortunately, these studies failed to document the occurrence of pollutant within the cytoplasm and the documented effects remain circumstantial and speculative. The present paper, instead, documents the presence and potential distribution of Hg within *A*. *parkinsoniana* cytoplasm and its possible cytological alterations.

Nile Red distinguished neutral lipids from polar ones (Fig 1A–1D) [39]. In our highest Hg-concentration, we found that more accumulation of neutral lipids occurred in the form of LD compared to lower Hg or controls. Lipid droplets have fundamental roles in a cell’s metabolism and are commonly related to the cell’s requirement to store excess lipid in neutral-lipid oils like triacylglycerols and sterol esters [40]. Increasing numbers of LD have been reported in lichen species (*Evernia prunastri* and *Xanthoria parietina*) as an effect of dust pollution from a cement industry [41] and in rat hepatocytes in response of Bisphenol A exposure [42]. An increase in the number of LD coupled with their dimension in the hepatocytes of grey mullet (*Mugil cephalus*) was related to heavy-metal pollution [43]. Similar results were documented in the hepatocytes of silver catfish (*Rhamdia quelen*) exposed to sublethal lead concentration [44], in *Daphnia similis* as a response to nanowire exposure [45] and in male rockfish (*Sebastiscus marmoratus*) after paclobutrazol exposure [46]. Decreasing numbers and dimensions of LD in the liver of fish (male guppies *Poecilia reticulata*) were associated with the addition of cysteine in their diet to reduce Hg concentration [47]. Increased number and size of lipid vesicles were detected in the liver of HgCl_2_-treated zebrafish (*Danio rerio*) and confirmed by the transcriptome analysis on the regulation of fatty acid synthesis and of mitochondrial fatty acid beta-oxidation [48]. Lipid droplets are also hypothesized to sequester toxicants in order to protect cells [49–50].

Our results suggest an increased number of LD associated with exposure to high Hg concentrations (100 ppm). This observation is supported by increased values of yellow fluorescence marking neutral lipid occurrence associated with higher concentrations of Hg. There was no statistically significant increase in the dimensions of lipids with respect to Hg except for significantly larger lipids in the T2-100 ppm treatment. It might be speculated that Hg-pollution might promote the proliferation of higher numbers of neutral lipids but not affect their dimension. Although based on a qualitative approach, [10] reported increased numbers and dimensions of lipid in two foraminiferal species (*Ammonia beccarii* and *Ammonia tepida*) due to Cu contamination. The exact implications and consequences of increases in LD numbers have not been explored in our present study.

All control specimens treated with AO were characterized by a weak, diffuse, green fluorescence of cytoplasm with some dispersed, low numbers and reduced dimension of red vesicles (lysosomes). Lysosomes, acidic membrane-bound organelles containing hydrolytic enzymes, are devoted to digestion of biomolecules, redundant or damaged organelles and proteins as part of autophagic cellular turnover [51–52]. A wide array of chemicals including metal ions (Fe, Cu and Hg), PAHs, nanoparticles can be effectively sequestered and accumulated by lysosomes [53] that might also represent the most important site of metal detoxification in eukaryotes [52].

Our AO results document that increased concentrations of Hg initially promote the proliferation of lysosomes both in terms of number and dimension. Increased production of lysosomes and lysosomal accumulation of Hg have been described in cells of insects (*Aedes albopictus*) and slugs (*Arion ater*) [54–55]. Lysosomal proliferation as a detoxification mechanism induced by metal contamination has been reported in mussels, oysters, marine dinoflagellate and foraminifera (i.e., [10, 56] and reference therein). More specifically, inorganic Hg after having formed a complex with selenium or cysteine was reported to accumulate in lysosomes in the liver of the frog *Rana ridibunda* [57]. The observed lysosomal compartment enlargement is commonly associated with the formation of secondary lysosomes (such as autolysosomes) and degradation process intensification, and may be related to Hg-dependent cell damage [58]. Curiously, at the highest Hg concentrations in our study, lysosomal membrane destabilization likely induced the acidification of chambers early in the experiment (T1-100 ppm; Fig 2C) whereas the very low levels of lysosomal activity manifest as AO MFI that occurred at the end of the experiment (T2-100 ppm) may have been due to specimen death. Pollutants are known to destabilize the membranes of lysosomes causing the release of hydrolytic enzymes [59], as reported in eukaryotes [60] including protists [61].

## Conclusion

This study examined the effect and bioaccumulation of Hg on a benthic foraminiferal species. Our experiment coupled with different microscopy techniques revealed, for the first time, the presence of Hg in the cytoplasm of *A*. *parkinsoniana* after exposure to elevated Hg in seawater. Our observations show that: (i) an increase in the number of LD, which have been reported as a detoxifying organelle where pollutants might be sequestered and biologically inactivated, in response to Hg exposure; (ii) Hg appears to promote the proliferation of lysosomes both in terms of number and dimension up to membrane destabilization when the lysosomal contents likely were released into the cytosol ultimately leading to cell death; (iii) *A*. *parkinsoniana* appeared to accumulate Hg in the organic lining at the basal part of pores and in the foramen/septa as well as in cytoplasm, mainly in the younger chambers.

## Supporting Information

S1 Appendix Lipid dimension counting. (XLSX)

S2 Appendix Mann-Whitney U Test for differences lipid dimension. Significant differences are marked in bold.
(XLS)

S1 Fig Typical paired NR confocal images. (A) Bright Field image. (B) Neutral lipids (triglycerides, esters of cholesterol and free fatty acids) in yellow. (C) Polar lipids (phospholipids, sphingolipids and non-esterified cholesterol) in red. (D) Merged Bright Field, yellow and red channels.
(TIF)

S2 Fig (A) Secondary emission image of a microtomed sample, organic lining (arrows), deposited on freshly peeled mica and coated with 10 nm of carbon to prevent sample charging. Included is a net elemental cross-section with a 10×10 pixel average of the sample from EDS mapping at 20 kV. (B) Elemental cross-sections showing the increase of Hg in the brighter region of the SEM image. The presence of Al, Si, K, and O comes primarily from the underlying mica (muscovite KAl_2_(AlSi_3_)O_10_(OH)_2_). (C) EDS spectra from the entire SEM image region (shown in A) showing the presence of Hg. (D) EDS maps of the SEM image region showing the depletion of carbon and the localized presence of Hg. The EDS maps are taken from the net intensities of the element regions. Each point is the average of 3×3 pixels.
(TIF)
