# Supplementary figures and images for: Mercury-Pollution Induction of Intracellular Lipid Accumulation and Lysosomal Compartment Amplification in the Benthic Foraminifer Ammonia parkinsoniana
Source: PLoS One. 2016 Sep 7;11(9):e0162401. doi: 10.1371/journal.pone.0162401 (PMC5014445; doi:10.1371/journal.pone.0162401)

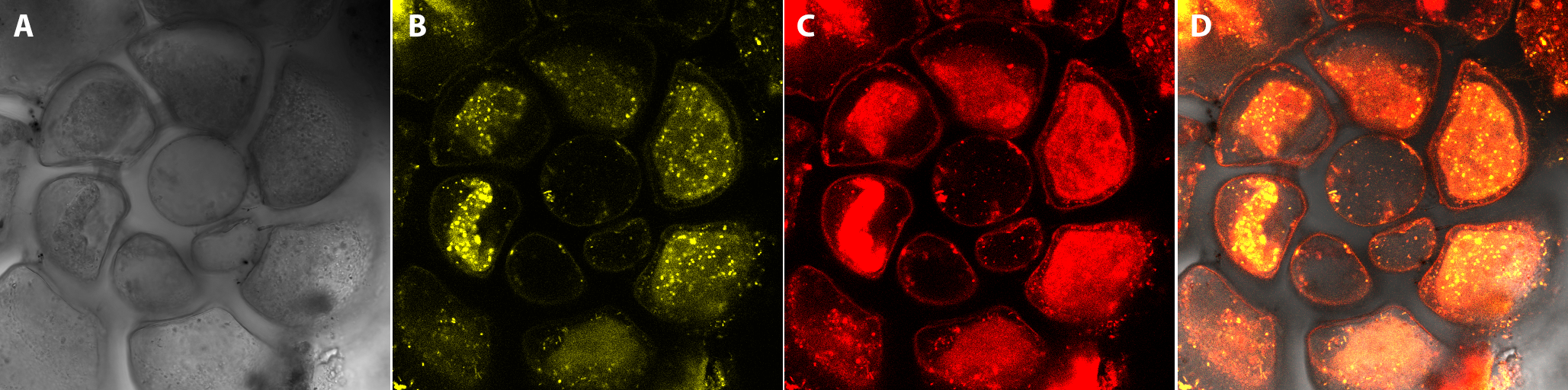

Supplement: S1 Fig — (A) Bright Field image. (B) Neutral lipids (triglycerides, esters of cholesterol and free fatty acids) in yellow. (C) Polar lipids (phospholipids, sphingolipids and non-esterified cholesterol) in red. (D) Merged Bright Field, yellow and red channels. (TIF) [file pone.0162401.s003.tif]

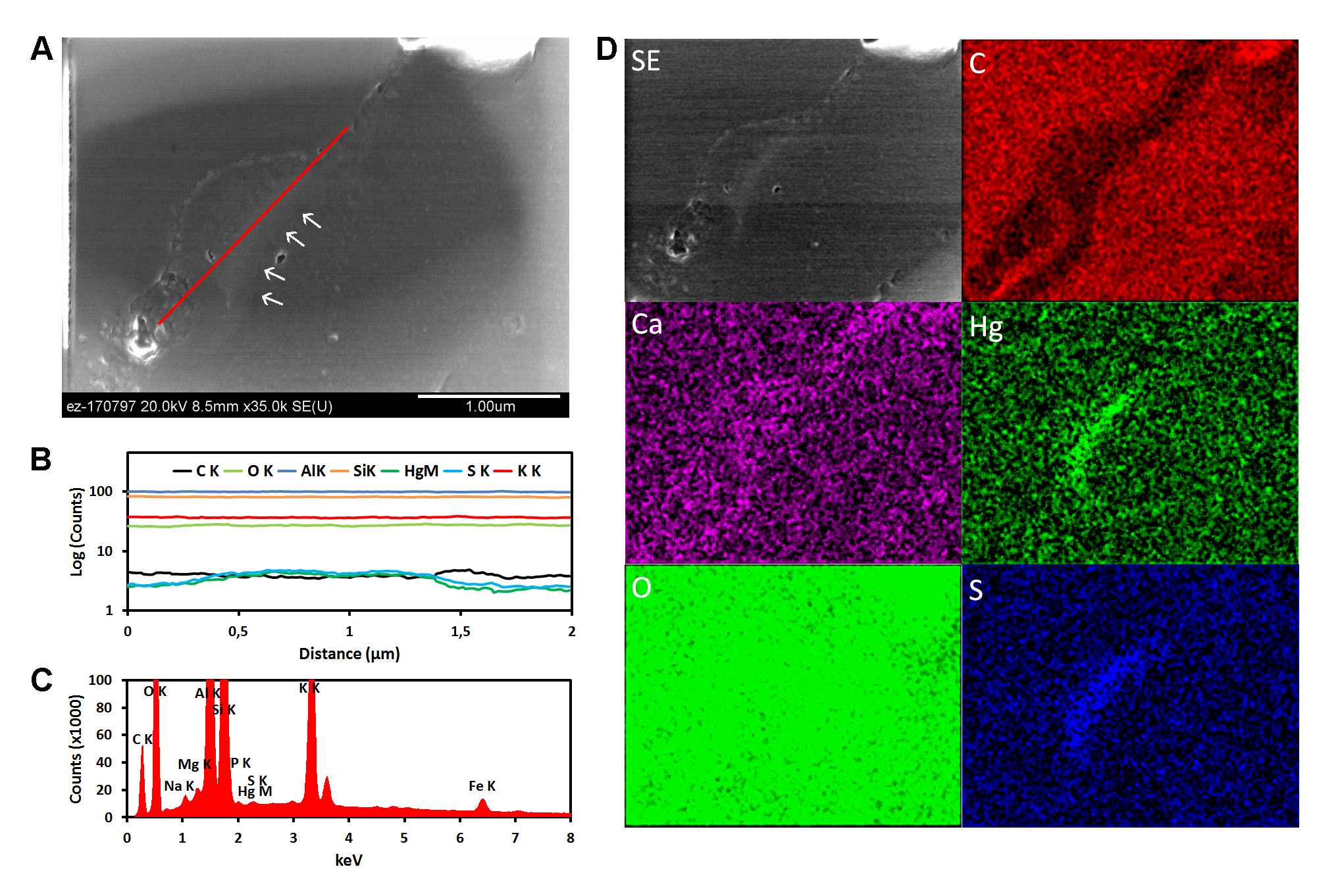

Supplement: S2 Fig — (A) Secondary emission image of a microtomed sample, organic lining (arrows), deposited on freshly peeled mica and coated with 10 nm of carbon to prevent sample charging. Included is a net elemental cross-section with a 10×10 pixel average of the sample from EDS mapping at 20 kV. (B) Elemental cross-sections showing the increase of Hg in the brighter region of the SEM image. The presence of Al, Si, K, and O comes primarily from the underlying mica (muscovite KAl2(AlSi3)O10(OH)2). (C) EDS spectra from the entire SEM image region (shown in A) showing the presence of Hg. (D) EDS maps of the SEM image region showing the depletion of carbon and the localized presence of Hg. The EDS maps are taken from the net intensities of the element regions. Each point is the average of 3×3 pixels. (TIF) [file pone.0162401.s004.tif]
